# Efficient Adsorption Removal of Phosphate from Rural Domestic Sewage by Waste Eggshell-Modified Peanut Shell Biochar Adsorbent Materials

**DOI:** 10.3390/ma16175873

**Published:** 2023-08-28

**Authors:** Cancan Xu, Rui Liu, Lvjun Chen, Quanxi Wang

**Affiliations:** 1 School of Environmental and Geographical Sciences, Shanghai Normal University, Shanghai 200234, China; xucancan523127@126.com; 2 Zhejiang Provincial Key Laboratory of Water Science and Technology, Department of Environment, Yangtze Delta Region Institute of Tsinghua University, Jiaxing 314006, China; chenlj@tsinghua.edu.cn; 3 School of Environment, Tsinghua University, Beijing 100084, China; 4 College of Life Science, Shanghai Normal University, Shanghai 200234, China

**Keywords:** phosphate adsorption, modified biochar adsorbent materials, rural domestic sewage, discharge standard

## Abstract

In order to promote the improvement of the rural living environment, the treatment of rural domestic sewage has attracted much attention in China. Meanwhile, the rural regions’ sewage discharge standards are becoming increasingly stringent. However, the standard compliance rate of total phosphorus (TP) is very low, and TP has become the main limiting pollutant for the water pollutants discharge standards of rural domestic sewage treatment facilities. In this study, waste eggshell (E) was employed as a calcium source, and waste peanut shell (C) was employed as a carbon source to prepare calcium-modified biochar adsorbent materials (E-C). The resulting E-C adsorbent materials demonstrated efficient phosphate (P) adsorption from aqueous solutions over the initial pH range of 6–9 and had adsorption selectivity. At an eggshell and peanut shell mass ratio of 1:1 and a pyrolysis temperature of 800 °C, the experimental maximum adsorption capacity was 191.1 mg/g. The pseudo second-order model and Langmuir model were best at describing the adsorption process. The dominant sorption mechanism for P is that Ca(OH)_2_ is loaded on biochar with P to form Ca_5_(PO_4_)_3_OH precipitate. E-C was found to be very effective for the treatment of rural domestic sewage. The removal rate of TP in rural domestic sewage was 91–95.9%. After adsorption treatment, the discharge of TP in rural sewage met the second-grade (TP < 3 mg/L) and even first-grade (TP < 2 mg/L). This study provides an experimental basis for efficient P removal by E-C adsorbent materials and suggests possible applications in rural domestic sewage.

## 1. Introduction

China is a big agricultural country with nearly 500 million rural people. Due to population growth and the improvement in social and economic living standards, the increase in rural domestic sewage is becoming one of the main environmental problems in rural areas [1]. In recent years, in order to reduce pollutant emissions and improve the rural living environment, rural domestic sewage treatment has received more and more attention by the Chinese government. Meanwhile, rural areas’ sewage discharge standards are becoming more and more stringent [2]; currently, thirty-one provinces and cities have issued rural sewage discharge standards in China.

The most conventional treatment methods for domestic sewage in rural regions consist of an operational sequence of aerobic/anoxic/aerobic processes. Anaerobic–anoxic–oxic (A^2^O) is one of the most typical schemes in China. However, some reports have observed that the efficient removal of COD, NH_3_-N, and total phosphorus (TP) is hard to realize [3]. In the study by Yang et al., surrounding Erhai Lake in Yunnan Province, centralized rural sewage treatment facilities had good removal efficiency. However, some centralized sewage treatment facilities could not meet the water pollution discharge standards, in particular, the average standard compliance rate of TP was only 4.26%. TP has become the main limiting pollutant for the discharge standards of rural domestic sewage treatment facilities. Therefore, improving TP treatment efficiency for meeting discharge standards in rural regions is an important target of future research [4].

Different biological, chemical, and physical techniques have been created and widely applied in previous studies to manage and treat the phosphorus (P) present in wastewater [5,6]. Among these methods, the adsorption method is the most attractive since it is economically feasible and simple in real application. In addition, the nutrient-loaded adsorbents can be further utilized as a soil conditioner and fertilizer [7,8]. Therefore, it is an important research topic to prepare cost-effective adsorbents with good adsorption performance. Recently, the modified biochar synthesized from calcium-rich waste achieved a good P adsorption effect. For example, when marble waste was employed as a Ca source, agricultural waste was used as a carbon source to synthesize modified-biochar composites to remove P from waste streams, and the maximum P removal capacity of the composites was 263.17 mg/g. The methods of producing modified biochar are economical, environmentally friendly and green [9]. Tobacco straw was employed as the carbon source, while oyster shell was used as the source of Ca to create a modified-biochar adsorbent with a maximum P adsorption capacity of 88.64 mg/g [10]. According to statistics, the crop straw output was 819.7 tons, of which peanut shell straw, wheat straw and corn straw accounted for 8.9%, 21.6% and 28.9% in China, respectively [11]. Most peanut shells are disposed of by landfill or incineration, resulting in an impact on the environment and the waste of resources. The preparation of biochar from peanut shells is a low-cost, sustainable, win–win and green production method that can reduce peanut shell’s environmental pollution [12]. Pure biochar is effective at adsorbing organic pollutants and cations [13,14], but the P removal rate is low by biochar from unmodified peanut shell waste, and modified biochar by metal is usually chosen to achieve P adsorption [11,15]. Eggshells contain calcium carbonate (~94%) and organic matter (~6%) [16]. By 2018, the world had generated approximately 8.6 million metric tons of eggshell waste [17]. Additionally, eggshells have the potential to be a source of calcium for the creation of Ca-modified biochar, which has the ability to absorb P and then be used as a phosphate fertilizer [16].

The removal of P in wastewater from pig farm, cattle farm, pond and human urine by Ca-modified biochar has been reported [9,10]. So far, the reports on the P treatment of rural domestic sewage by Ca-modified biochar are limited. In this study, in order to improve TP treatment efficiency to meet discharge standards in rural regions, waste eggshell was used as a Ca source and peanut shell waste as a carbon (C) source to synthesize adsorbent material (E-C) by pyrolysis. Some adsorption models were used to observe the adsorption performance of P by E-C. The mechanisms of P adsorption on the prepared E-C were analyzed. The feasibility of E-C as an adsorbent for TP removal was also assessed using rural domestic sewage. This study combines agricultural waste recycling with phosphorus pollution control, and we provide a possibility scheme for effective phosphorus removal/recovery from rural domestic sewage.

## 2. Materials and Methods

### 2.1. Experimental Materials

The peanut shells were obtained locally in Jiaxing (China). Before usage, they were air-dried and then ground to pass through 40 mesh sieves (0.425 mm). Waste eggshells were obtained from a mess hall in Jiaxing. They were dried at 60 °C in an oven after being cleaned three times. Additionally, there were then ground to pass through 40 mesh sieves (0.425 mm) before use. All chemical reagents (such as KH_2_PO_4_, HCl, NaOH, KCl, KNO_3_, K_2_SO_4_ and KHCO_3_) were of analytical grade and purchased from Aladdin Industrial Corporation (Shanghai, China).

### 2.2. Preparation of Adsorbents

The predetermined weights of the eggshell and peanut shell power were determined in a mass ratio of 1:1. Deionized water was then added to the mixture, and then the mixture was blended overnight with a magnetic mixer. The mixture was placed in a 105 °C oven to dry. The dried mixture was transferred to a quartz boat, and then the mixture was placed in the tube furnace and calcined for 2 h under a nitrogen atmosphere at 800 °C. The heating rate was set at 5 °C/min. A pure peanut shell prepared biochar was named BC.

### 2.3. Phosphorus Adsorption Experiments

In this study, all adsorption experiments were carried out in batches. E-C adsorbent materials were placed in a 100 mL glass vial with a stopper containing 40 mL of P solutions. The vials were oscillated (180 rpm) in a shaker bath (HZQ-F100 Taicang, China) at pH 7 and 25 °C. The E-C adsorbent dose was 250 mg/L. Then, the suspension was filtered through a 0.45 μm PES syringe filter, and the filtered solution was used to detect the phosphorus concentration. A total of 0.1 mol/L NaOH or HCl was used to adjust the initial pH values of the solutions. KH_2_PO_4_ was used as a phosphorus source in the solutions because the main form of phosphate in the range of 2.13–7.20 is H_2_PO_4_^−^ [18]. All experiments were performed in triplicate.

To evaluate the equilibrium adsorption parameters, adsorption isotherm experiments were conducted as follows: E-C dose, 250 mg/L; initial P concentrations of 5, 10, 25, 50, 100, 150 and 200 mg/L; initial pH, 7.0; and adsorption time, 24 h.

Adsorption kinetic tests were conducted as follows: E-C dose, 250 mg/L; initial P concentration, 100 mg/L; initial pH, 7.0; and adsorption time, 1–1440 min.

The effect tests of common competing substances (Cl^−^, NO_3_^−^, SO_4_^2−^ and HCO_3_^−^) on P removal by E-C were evaluated as follows: E-C dose, 250 mg/L; initial phosphate concentration, 100 mg/L; Cl^−^, NO_3_^−^, SO_4_^2−^ and HCO_3_^−^ concentrations, 100 and 1000 mg/L; and adsorption time, 24 h.

The effects of different initial pH values on P removal by E-C were evaluated as follows: E-C dose, 250 mg/L; initial P concentration, 100 mg/L; initial pH of 6.0, 7.0, 8.0 and 9.0; and adsorption time, 24 h.

Rural residential sewage from two centralized rural sewage treatment facilities in Jiaxing (Zhejiang province) were utilized to assess the E-C adsorbent’s suitability for actual phosphorus sewage. The first centralized rural sewage treatment facility was the anaerobic–anoxic–oxic (A^2^O) process (named as F1), and the design capacity was 50 t/d. The second centralized rural sewage treatment facility was an A^2^O process (named as F2), and the design capacity was 20 t/d. Every three days, a sample of rural home sewage was taken from the secondary sedimentation tank. A total of 40 mL of rural home sewage was collected in 100 mL glass vials with stoppers containing 0.01 g of E-C adsorbent materials, and the vials were oscillated (at 180 rpm) in a shaker bath for 4 h at 25 °C.

All experiments were repeated three times. The equilibrium adsorption capacity (q_e_, mg/g) and adsorption capacity at different times t (q_t_, mg/g) was calculated as follows:

q_e_ = (C_0_ − C_e_) V/m (1)

q_t_ = (C_0_ − C_t_) V/m (2)

where C_0_ (mg/L) represents the initial P concentration, C_e_ (mg/L) represents the P concentration at equilibrium, and C_t_ (mg/L) represents the P concentration at time t; V (L) is the volume of the solution; and m (g) is the adsorbent mass.

### 2.4. Characterization and Analytical Method

The concentration of phosphorus in solution was determined using ammonium molybdate spectral photometry using an ultraviolet spectrophotometer (SHIMADZU UV-2450, Kyoto, Japan) according to the standard method [11]. The surface elements and morphologies of adsorbents before and after adsorption of P were studied using scanning electron microscopy with energy dispersive X-ray spectroscopy (SEM-EDS) (Regulus-810, Hitachi, Tokyo, Japan). The diffraction patterns of adsorbents before and after P adsorption were studied using an X-ray diffractometer (XRD, Bruker D8 Advance X-ray diffractometer, Karlsruhe, Germany). An FTIR spectrometer (Thermo Scientific Nicolet IS50, Waltham, MA, USA) was used to study the characterization of the functional groups in the adsorbents before and after P adsorption. A fully automated physisorption instrument (JW-BK200B, Beijing, China) was used to detect the specific surface area, pore volume, and average pore size of the adsorbents.

## 3. Results and Discussion

### 3.1. Basic Physicochemical Properties of Biochar

By weighing the samples before and after pyrolysis at 800 °C, the resulting mass loss (49.3%) during the preparation of the E-C adsorbent material was achieved. The basic physicochemical properties and major elements of the E-C and BC adsorbent materials are shown in Table 1. BC showed a specific surface area of 9.203 m^2^/g, average pore size of 2.95 nm, and pore volume of 0.083 cm^3^/g; the corresponding values for E-C were 64.956 m^2^/g, 6.46 nm and 0.105 cm^3^/g, respectively.

The eggshell decomposed into CO_2_ and CaO during high-temperature pyrolysis [19]. The formation of CO_2_ broadens the material’s pore size as an activation substance, and it can therefore be used to produce activated carbon [20]. In addition, the adsorbent’s pore volume and specific surface area may increase by adding eggshell to the biochar [21].

The pore volume and surface area of the E-C were higher than those of the BC. Biochar’s adsorption performance largely depends on its porosity and specific surface area. The larger the adsorbent’s specific surface area and higher the porosity, the more surface adsorption sites there will be and the higher the adsorption performance [10,22,23]. After 24 h of adsorption, the P adsorption capacity of BC was only 5.3 mg/g, but the E-C reached 191.1 mg/g. This showed that Ca from CaCO_3_ in eggshells was successfully introduced into the E-C adsorbent materials.

### 3.2. Adsorption Kinetics

**Figure 1:**
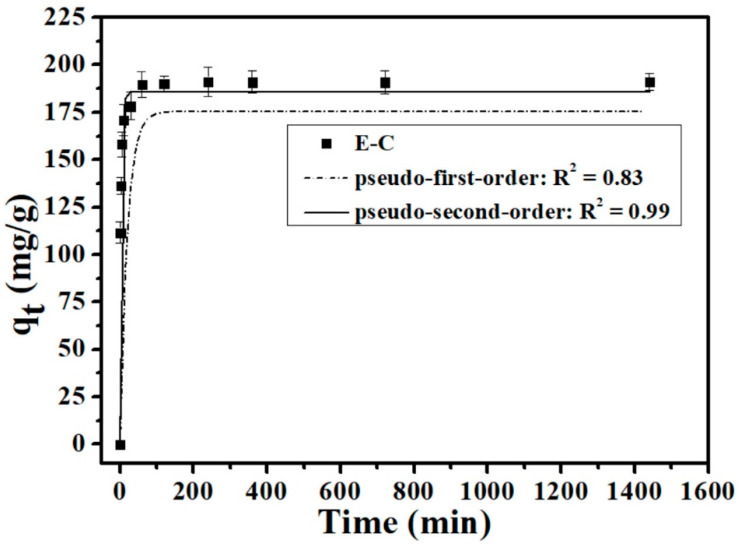
P adsorption kinetics onto E-C (dosage: 250 mg/L; temperature: 25 °C; initial P concentration: 100 mg/L; and pH = 7).

Figure 1 shows the kinetic behavior results of P onto the E-C adsorbent materials. Clearly, during the initial hour the adsorption processes of E-C for P was rapid, with the rate slowing down until reaching an equilibrium within 4 h. The adsorption process involves the initial rapid diffusion of ions from the solution onto the surface of the external adsorbent. This is then followed by a slow adsorption process through the diffusion of ions into the pores on the inner surfaces of the adsorbent [20].

The pseudo-first-order model and pseudo-second-order model were used in the study of E-C adsorbent materials’ adsorption kinetics for P in aqueous solutions:

Pseudo-first-order kinetic equation: q_t_ = q_e_(1 – e^−k^_1_^t^) (3)

Pseudo-second-order kinetic equation: t/q_t_ = 1/k_2_q_e_^2^ + t/q_e_ (4)

where q_t_ is the P adsorption amounts at time t, and q_e_ is the P adsorption amounts at equilibrium time; k_1_ is the first-order kinetics’ adsorption rate constant, and k_2_ is the second-order kinetics’ adsorption rate constant. The results showed that the coefficient of determination simulated with the pseudo-second-order model (*R*^2^ = 0.99) is higher than the pseudo-first-order model (*R*^2^ = 0.83) (Figure 1), which suggested that the pseudo-second-order kinetic model can well describe the adsorption kinetic process of P onto E-C, and a monolayer chemisorption occurred on the adsorbent surface. Based on the analysis of the pseudo-second-order theory and the chemisorption characteristics of E-C adsorbent materials, chemisorption was the main rate-limiting step of P adsorption by E-C [10,24].

### 3.3. Adsorption Isotherms

In this study, the isothermal experiments were carried out with different initial P concentrations (5, 10, 25, 50, 100, 150 and 200 mg/L). Langmuir and Freundlich adsorption isotherm models were used in the study of E-C adsorbent’s adsorption isotherms for P in aqueous solutions:

Langmuir: q_e_ = K_L_q_max_C_e_/(1 + K_L_C_e_) (5)

Freundlich: q_e_ = K_F_C_e_^1/n^ (6)

where q_e_ (mg/g) is the equilibrium adsorption amount, K_L_ (L/mg) is the Langmuir adsorption equilibrium constant, q_max_ (mg/g) represents the maximum adsorption capacity, C_e_ (mg/L) represents the P equilibrium concentration, K_F_ (mg^(1−1/n)^L^1/n^/g) represents the Freundlich adsorption constant representing the adsorption capacity of the adsorbent materials, and n represents an indication of linearity.

**Figure 2:**
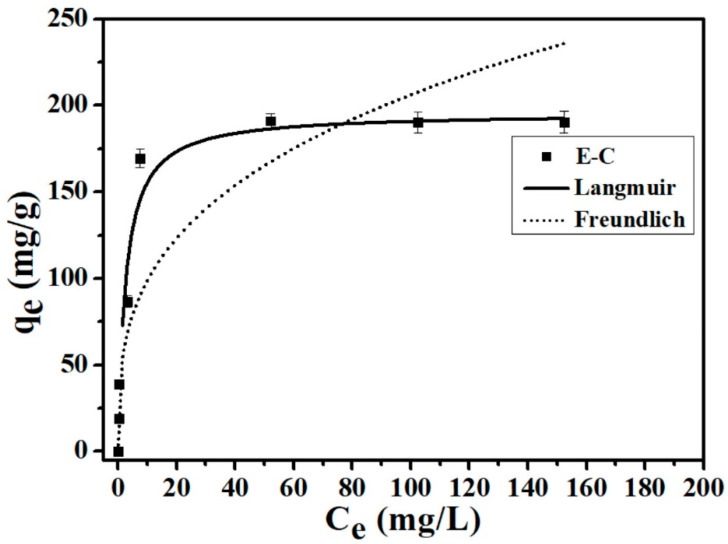
P adsorption isotherms onto the E-C (dosage: 250 mg/L; temperature: 25 °C; adsorption time: 24 h; and pH = 7).

The fitted adsorption isotherms of P onto the E-C adsorbent materials are shown in Figure 2. The fitting effect of the Langmuir model on the experimental data is better than that of the Freundlich model in this study (Figure 2). The corresponding fitting parameters for the Langmuir model are as follows: q_max_ = 195.8 mg/g, K_L_ = 0.39 L/mg, and *R*^2^ = 0.95. The corresponding fitting parameters for the Freundlich model are as follows: K_F_ = 47.3 mg^(1−1/n)^L^1/n^/g, 1/n = 0.32 L/mg, and *R*^2^ = 0.91. Furthermore, the regression coefficient *R^2^* of the Langmuir model is higher than that of the Freundlich model. it is further confirmed that the Langmuir isotherm model adequately describes the experimental data of P adsorption by E-C, implying that the effective adsorption surface of E-C is homogeneous and a monolayer during the adsorption of P. this is consistent with the above adsorption kinetic results described by the pseudo-second-order model. Similar P adsorption behavior has been reported in other research of Ca-based adsorbents [10,25,26,27,28].

The q_max_ of the E-C, through calculating the Langmuir isotherm, was 195.8 mg/g, which was consistent with the experimental maximum adsorption capacity of 191.1 mg/g, indicating that the E-C adsorbent materials in aqueous solution had excellent adsorption properties for removing P. The E-C adsorbent material’s adsorption capacity for P was higher than that of the Ca-biochar adsorbent prepared from eggshell and peanut shells through ball milling and chemical impregnation methods (the maximum P adsorption capacity was 130.57 mg/g) reported by Liu et al. [28].

### 3.4. Effect of Coexisting Anions

**Figure 3:**
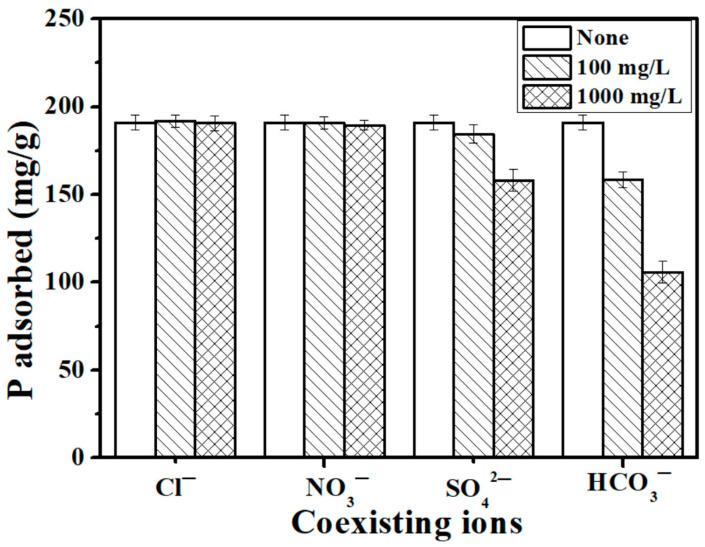
Effect of coexisting ions (Cl^−^, NO_3_^−^, SO_4_^2−^, and HCO_3_^−^) on the P adsorption capacity of E-C (dosage: 250 mg/L; temperature: 25 °C; adsorption time: 24 h; initial P concentration: 100 mg/L).

In natural water and wastewater, some common anions are present that may compete with P for adsorption sites on the adsorbent [29]. Therefore, to further explore the adsorption ability of E-C, coexisting ions (i.e., KCl, KHCO_3_, KNO_3_ and K_2_SO_4_) were added into the adsorption system. These common anions have different levels of effect on the P adsorption capacity of the E-C adsorbent materials, following the order HCO_3_^−^ > SO_4_^2−^ > NO_3_^−^ > Cl^−^ (Figure 3). The results showed that the anions NO_3_^−^ and Cl^−^ had limited effect on the adsorption process of P; even for ion concentrations up to 1000 mg/L for Cl^−^ and NO_3_^−^, the adsorption capacity of P remained at 190.6 mg/g and 189.5 mg/g, respectively. The SO_4_^2−^ anions had some effect on the adsorption process of P. As the concentration of SO_4_^2−^ gradually increased from blank control to 100 mg/L and 1000 mg/L, the adsorption capacity of E-C decreased from 191.1 to 184.5 and 158.1 mg/g, respectively.

On the other hand, HCO_3_^−^ had a significant negative effect on the adsorption of P by E-C adsorbent materials. When the concentration of HCO_3_^−^ gradually increased, P adsorption of the E-C adsorbent materials had a significant downward trend. HCO_3_^−^ ionization in solution can generate CO_3_^2−^, and the adsorption sites on E-C are reduced because CO_3_^2−^ will compete with P to bind Ca to produce a precipitate. Similar results have been reported in the study of other calcium-rich phosphate adsorbents [20,30]. However, when the concentration of HCO_3_^−^ was as high as 1000 mg/L, P adsorption of E-C still remained at 105.8 mg/g. Thus, the E-C adsorbent materials have a higher adsorption selectivity for phosphate anions than for many other common anions. The above results show that the E-C adsorbent materials have significant potential in practical P-containing wastewater applications even in the presence of Cl^−^, NO_3_^−^, SO_4_^2−^, and HCO_3_^−^ common ions.

### 3.5. Influence of pH

**Figure 4:**
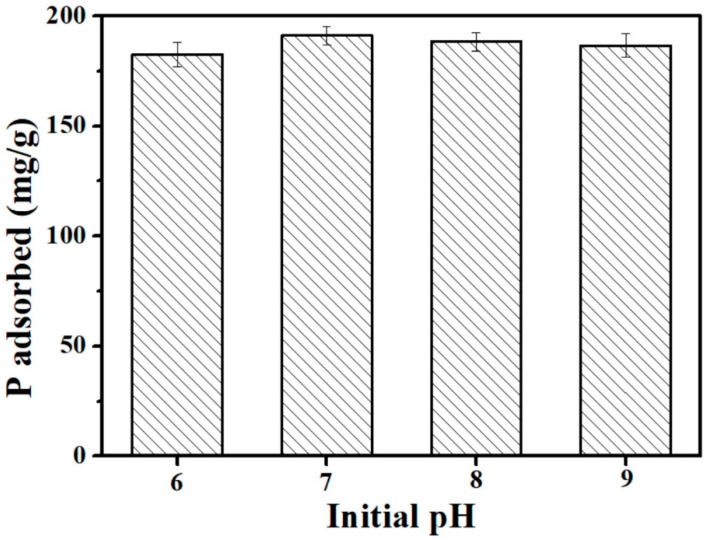
The effect of initial pH on the P adsorption capacity of the E-C (dosage: 250 mg/L; temperature: 25 °C; adsorption time: 24 h; and initial P concentration: 100 mg/L).

The effect of pH is an important parameter impacting the absorption performance of a sorbent. Since the pH of rural domestic sewage is generally nearly 6–9, the effect of the initial solution pH (6–9) on the adsorption of P onto the E-C was examined. The P adsorption amount onto the E-C increased from 182.6 mg/g at pH = 6 to 191.1 mg/g at pH = 7 (Figure 4). Additionally, the P adsorption amount onto the E-C slightly decreased to 188.5 mg/g at pH = 8 and 186.7 mg/g at pH = 9 (Figure 4). Thus, the E-C adsorbent material showed a good adsorption effect on P under the initial pH range of 6–9.

In this study, calcium-active components contributed to the adsorption of P onto the E-C adsorbent material by strong chemical interaction mechanisms, resulting in the high adsorption capacity of the E-C for P over the pH range (6–9).

### 3.6. Adsorption Mechanisms

**Figure 5:**
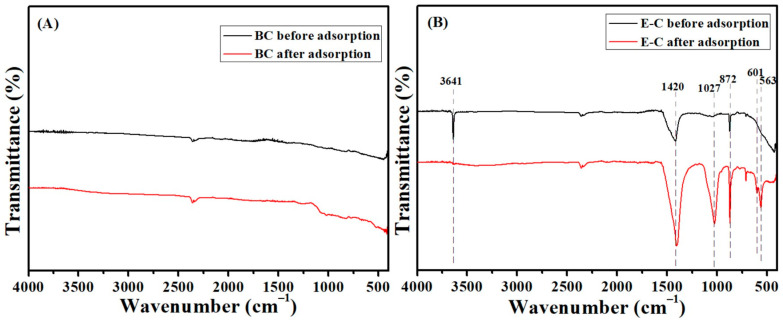
The FTIR spectra of BC (**A**) and E-C (**B**) before and after P adsorption.

The adsorbent materials before and after P adsorption were analyzed by XRD, SEM and FTIR, and the adsorption mechanisms of P onto the E-C adsorption materials were discussed. Before and after P adsorption, the characteristic peaks of the BC had no significant change in the FTIR spectra analysis (Figure 5A). This was attributed to the fact that the ingredients in the BC did not react chemically with phosphates. The weak P adsorption property of the BC might be related to physical adsorption. Small amounts of P might be adsorbed into the pores of the BC. However, before and after P adsorption, the characteristic peaks of the E-C had a significant change in the FTIR spectra analysis (Figure 5B). The peaks at 872 cm^−1^ (C-O) and 1420 (C=C) were observed for the adsorbent before and after adsorption, suggesting that the C-O and C=C groups did not contribute to the adsorption of P. Before adsorption, the peak at 3641 cm^−1^ (O-H) was attributed to Ca(OH)_2_ for the E-C [24]. After adsorption, the peak at 3641 cm^−1^ vanished, showing that the -OH took part in the chemical adsorption process of P. Furthermore, the peaks at 563, 601 and 1027 cm^−1^ (P-O) were significantly enhanced and attributed to phosphates, showing that P was adsorbed onto the E-C adsorbent materials. Similar results were reported by other researchers [9,20].

**Figure 6:**
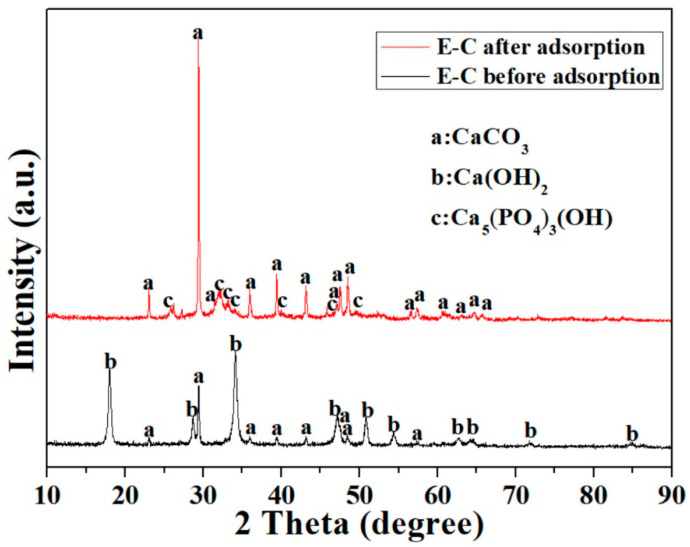
X-ray diffraction pattern of the E-C adsorbent materials before and after P adsorption.

The diffraction patterns of the E-C before and after P adsorption showed a significant change in the XRD spectra analysis (Figure 6). Before adsorption, the E-C had CaCO_3_ characteristic peaks (PDF #05-0586, 2*θ* = 23°, 29.4°, 36°, 39.4°, 47.5°, 48.5°, and 57.4°) and Ca(OH)_2_ characteristic peaks (PDF #04-0733, 2*θ* = 18.1°, 28.7°, 34.2°, 47.1°, 50.9°, 54.4°, 62.6°, 64.3°, 71.8°, and 84.8°). Ca(OH)_2_ groups are the main active sites in the E-C adsorbent materials for P adsorption. CaCO_3_ groups in eggshells were not fully decomposed at 800 °C. In Lee’s study, at a calcination temperature of 800 ℃, the mineral composition of eggshell still contained 35.7% CaCO_3_ [8].

After adsorption, Ca(OH)_2_ diffraction peaks vanished, and the E-C had Ca_5_(PO_4_)_3_OH characteristic peaks (PDF #09-0432, 2*θ* = 25.9°, 29°, 32.2°, 39.8°, 46.7°, 49.5°, 53.2°, and 64.2°). The Ca_5_(PO_4_)_3_OH, generated from the reaction of phosphate with Ca(OH)_2_, was responsible for the removal of P from the water, consistent with the FTIR and kinetic findings. In addition, the CaCO_3_ (PDF #05-0586) characteristic peaks still existed. It was pointed out that CaCO_3_ did not participate in the chemical reaction with P. CaCO_3_ might adsorb a small amount of P by physical adsorption.

**Figure 7:**
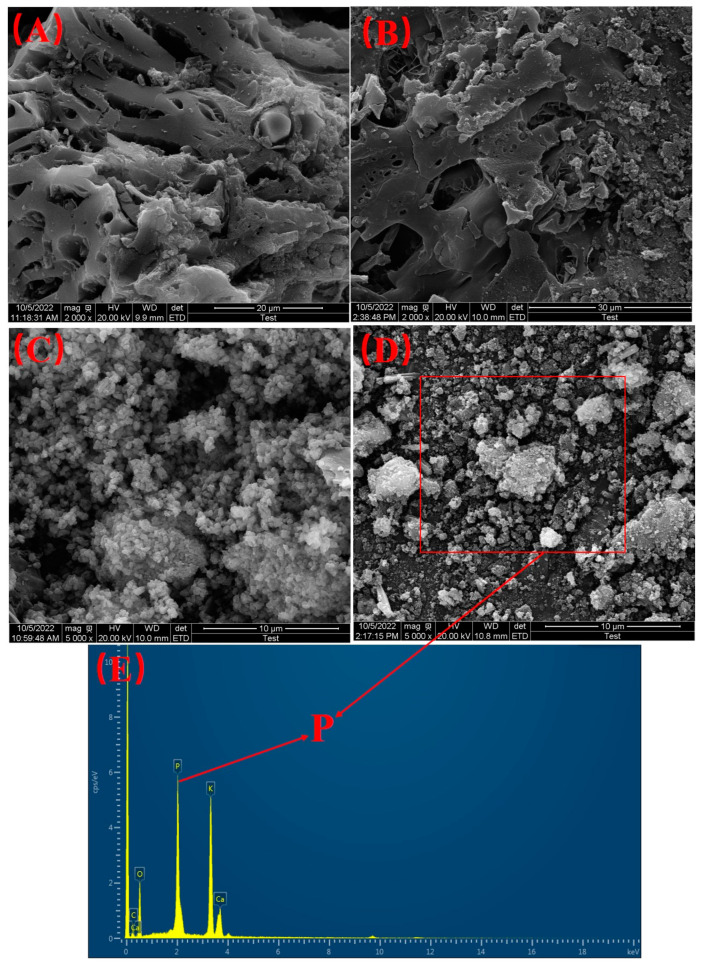
SEM photographs of the BC (**A**) before and (**B**) after P adsorption; E-C (**C**) before and (**D**) after P adsorption; (**E**) EDS image of the E-C after P adsorption.

Before and after P adsorption, the morphologies of the BC had no significant change though SEM images (Figure 7A,B). Before loading Ca, BC showed a porous structure, and SEM photography showed that the surface of the biochar was smooth, which might be related to the morphology of the biomass itself. The morphologies of the E-C adsorbent materials before and after P adsorption had a significant change when looking at the SEM images (Figure 7C,D). Before P adsorption, the surface of the E-C was relatively clean, but many impurity particles appeared on the surface. These particles were CaCO_3_ and Ca(OH)_2_. In addition, the E-C adsorbent materials had a porous structure before adsorption. After adsorption of P, a lot of flocculent precipitates appeared on the materials’ surfaces, and the pores of the E-C were blocked. The above results show that during the adsorption of P onto the E-C adsorbent materials, a good deal of flocculent precipitates were generated on the surface of the E-C adsorbent materials and in the pores. Therefore, the porosity and effective active sites of the E-C were reduced, and finally the adsorption saturation of the E-C adsorbent materials was induced. From Figure 7E, after the adsorption of P, the phosphorus element was distributed on the E-C. Therefore, combined with Figure 6, it can be inferred that the precipitates on the E-C should be Ca_5_(PO_4_)_3_OH.

According to the above results, the mechanisms of the E-C to adsorb P include physical adsorption, surface precipitation, and electrostatic attraction. The dominating sorption mechanism of the E-C for P is that Ca(OH)_2_ reacts with phosphate to form Ca_5_(PO_4_)_3_OH precipitates, and this result is similar to other studies [9,25,31].

### 3.7. Removal of Phosphate by E-C from Rural Domestic Sewage

**Figure 8:**
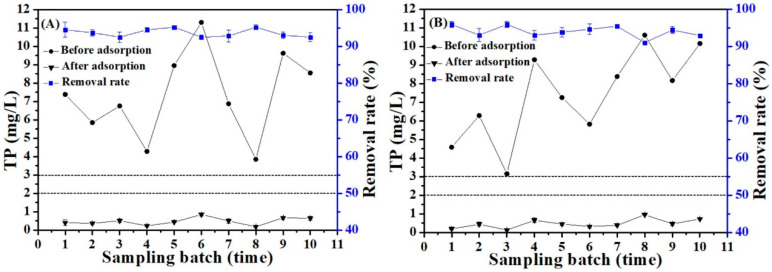
The removal rate of TP in rural domestic sewage by E-C, (**A**) F1 and (**B**) F2 (dosage: 250 mg/L; temperature: 25 °C; and adsorption time: 4 h).

It was observed from Figure 8 that the discharge of total phosphorus (TP) in rural domestic sewage of two centralized rural sewage treatment facilities in all sampling batches exceeded the second-grade Discharge Standard (DB33/973-2021) (TP < 3 mg/L) [32] in this study. The discharge of NH_3_-N, COD and pH in rural sewage of F1 was 12.1–22.8 mg/L, 42–85 mg/L and 6.8–7.2, respectively. The discharge of NH_3_-N, COD and pH in rural sewage of F2 was 6.8–19.1 mg/L, 35–76 mg/L and 6.7–7.2, respectively. The discharge of NH_3_-N, COD and pH in rural sewage of two centralized rural sewage treatment facilities met the second-grade Discharge Standard (DB33/973-2021) (COD < 100 mg/L, NH_3_-N < 25 mg/L, and pH 6~9) [32]. Phosphorus becomes the main limiting factor for reaching the discharge standard.

Figure 8 presents the results of phosphate adsorption by adding 0.01 g of E-C adsorbent materials to 40 mL of actual rural sewage. The TP content in the rural domestic sewage of F1 was 3.85–11.3 mg/L (Figure 8A), the TP content was 0.18–0.85 mg/L after adsorption, and the TP removal rate reached 92.5–95.2%. After adsorption treatment, the discharge of TP in rural sewage from all the sampling batches met the second-grade Discharge Standard (DB33/973-2021) (TP < 3 mg/L), even meeting the first-grade Discharge Standard (DB33/973-2021) (TP < 2 mg/L) [32].

The TP content in the rural domestic sewage of F2 was 3.15–10.6 mg/L (Figure 8B), the TP content was 0.13–0.95 mg/L after adsorption, and the TP removal rate reached 91–95.9%. After adsorption treatment, the discharge of TP in rural sewage from all the sampling batches met the second-grade Discharge Standard (DB33/973-2021) (TP < 3 mg/L), even meeting the first-grade Discharge Standard (DB33/973-2021) (TP < 2 mg/L).

Many complex components may be present in actual wastewater which can interfere with P removal by the adsorbent. In this study, the E-C adsorbent materials also exhibited a high P adsorption capacity from actual rural sewage. Therefore, the E-C adsorption materials have great potential in the treatment of large-scale rural domestic sewage containing P.

The recovery of adsorbents is an important economic parameter. However, the operation procedure of recycling P adsorbents is very complicated [21]. The main components of the E-C biochar materials are O, C, Ca and H; these elements are environmentally friendly, and the adsorbents have a mesoporous structure. After adsorption of P, a good deal of P-rich Ca_5_(PO4)_3_(OH) is produced on the E-C adsorbent materials; furthermore,, it also has the physical properties of biochar. Therefore, the P-adsorbed E-C adsorbent materials may be further used as soil regulators and fertilizer. This method can realize the reuse of E-C and realizes the virtuous cycle of P resources in the ecosystem [9,20,30,33].

## 4. Conclusions

In this study, Ca-modified biochar (E-C) adsorbent materials were prepared utilizing eggshell and peanut shell waste as raw materials. The E-C adsorbent materials were used to remove P from aqueous solutions, and the results show that the prepared E-C adsorbent materials exhibited an excellent adsorption capacity over the initial pH range of 6–9 and adsorption selectivity. An experimental maximum adsorption capacity of 191.1 mg/g was obtained by the E-C sample that was prepared with a mass ratio of 1:1 and a pyrolysis temperature of 800 °C. The dominant sorption mechanism for phosphate was due to Ca(OH)_2_ on the E-C adsorption materials reacting with phosphate to generate Ca_5_(PO_4_)_3_OH precipitate. E-C effectively removed P of rural domestic sewage. The removal rate of TP in rural domestic sewage was 91–95.9%. After adsorption treatment, the discharge of TP in rural sewage met second-grade Discharge Standard (DB33/973-2021) (TP < 3 mg/L), and even met the first-grade Discharge Standard (DB33/973-2021) (TP < 2 mg/L). This study provides the experimental basis for the efficient removal of phosphorus by E-C adsorbent materials, and it provides the possibility for its application in rural domestic sewage.

## Figures and Tables

**Table 1:** Specific surface area, pore volume, average pore size and major elements of E-C and BC.

Adsorbent	Physical Properties			Major Elements		
	Specific Surface Area (m^2^/g)	Pore Volume (cm^3^/g)	Average Pore Size (nm)	C (%)	O (%)	Ca (%)
E-C	64.956	0.105	6.46	33.23	45.1	21.3
BC	9.203	0.083	2.95	78.5	13.53	1.26

## Data Availability

Not applicable.
